# Coping With COVID-19: Emergency Stress, Secondary Trauma and Self-Efficacy in Healthcare and Emergency Workers in Italy

**DOI:** 10.3389/fpsyg.2020.566912

**Published:** 2020-09-03

**Authors:** Monia Vagni, Tiziana Maiorano, Valeria Giostra, Daniela Pajardi

**Affiliations:** Department of Humanities, University of Urbino, Urbino, Italy

**Keywords:** COVID-19, stress, secondary trauma, healthcare workers, self-efficacy, coping

## Abstract

Coping with the coronavirus disease (COVID-19) is a significant risk factor for the psychological distress of health workers. Hence, this study explores the relationship between coping strategies used by healthcare and emergency workers in Italy to manage the stress factors related to the COVID-19 emergency, which may result in the risk of developing secondary trauma. We study differences between healthcare (*n* = 121) and emergency workers (*n* = 89) in terms of their coping strategies, emergency stress, and secondary trauma, as well as the relationships of these differences to demographic variables and other stress factors (Instructions and Equipment). For this purpose, we collected data from participants through the following questionnaires online: *Secondary Traumatic Stress Scale – Italian Version*, *The Coping Self-Efficacy Scale – Short Form*, an original questionnaire on stressors, and the *Emergency Stress Questionnaire* (to assess organizational–relational, physical, decisional inefficacy, emotional, cognitive, and COVID-19 stress). We performed a *t*-test, correlational analysis, and hierarchical regression. The analyses reveal that compared with the emergency worker group, the health worker group has greater levels of emergency stress and arousal and is more willing to use problem-focused coping. Healthcare workers involved in the treatment of COVID-19 are exposed to a large degree of stress and could experience secondary trauma; hence, it is essential to plan prevention strategies for future pandemic situations. Moreover, individual efficacy in stopping negative emotions and thoughts could be a protective strategy against stress and secondary trauma.

## Introduction

The coronavirus disease (COVID-19), or the acute respiratory disease caused by severe acute respiratory syndrome coronavirus 2 (SARS-CoV-2), began spreading in China at the end of 2019 and, to date, represents an international health emergency without precedents in terms of its health, economic, and organizational effects on people’s lives (World Health Organization, 2020). After China, Italy was the first country to be affected by this epidemic, with the first deaths on February 20, 2020, and a rapid increase in the spread of infection and mortality. COVID-19 was first detected in Northern Italy, and it then spread, although at different rates of incidence, to the other regions. It was immediately evident that healthcare and emergency workers were at great risk of contagion and that protection and intervention protocols needed to be introduced in the absence of adequate points of reference because of the exceptional nature of the epidemic, the rate of spread of the infection, the seriousness of patients’ health condition, and the mortality index. The extreme conditions in which health workers have had to work, especially in the most affected regions in Northern Italy, are indicated by the following data from the Italian National Institute of Health (2020): over 150 doctors died and 25,000 other health workers were infected within the general context of the population of 30,000 deaths and 220,000 infections in a span of 11 weeks. It was also clear that the medical staff would experience serious psychological repercussions because of the working conditions as well as the difficulty of having scientific points of reference on care and intervention procedures. To this must be added the increase in workload, the extension of working hours and, for health workers, the frequent exposure to the suffering and death of their patients. Therefore, healthcare and emergency workers were subjected to serious psychological as well as physical stress. Hence, the aim of this study, which was also the aim of a previous study (Vagni et al., 2020), is to focus on the similarities and the differences in the stress management of two professional groups—healthcare and emergency workers—during the acute phase of the pandemic. Both groups have had to deal with COVID patients as frontline responders and have been exposed to the related risks of infection and psychological consequences, which, to date, have not been examined in detail through a comparative analysis.

As regards the stress that they experience, the literature clearly explains that healthcare and emergency workers who intervene in emergency situations are exposed to the risk of developing dysfunctional reactions that can be identified at different levels—physical and/or physiological (e.g., psychosomatic disorders, sleep/wake cycle alterations, and sense of tiredness); emotional (e.g., irritability, nervousness, agitation, anger, low self-esteem, and guilt); cognitive (e.g., distractibility, sense of ineffectiveness, and negative anticipation of events); and relational (e.g., increase in conflicts within emergency teams and/or with their organization/institution, and social withdrawal)—and may also develop reactions from secondary trauma (Del Missier et al., 2008; Sbattella, 2009; Argentero and Setti, 2011; Fraccaroli and Balducci, 2011; Bellelli and Di Schiena, 2012; Walton et al., 2020). Faced with stressful events regarding which they lack previous experience and specific, necessary knowledge, and which cause tension owing to the need for rapid decision timings and a sense of responsibility, emergency workers may experience a sense of decision ineffectiveness. In fact, emergency situations are characterized by high levels of decisional and operational uncertainty with associated regret and guilt (Del Missier et al., 2008).

Several studies have highlighted that insufficient instructions and a lack of personal protective equipment (PPE) are important predictors of stress for healthcare and emergency workers in large-scale emergencies (Oh et al., 2017; Du et al., 2020; El-Hage et al., 2020; Walton et al., 2020). Oh et al. (2017) highlighted that nurses involved in managing the Middle East respiratory syndrome (MERS) experienced lower levels of stress when the levels of goods supply and hospital training were higher. Some studies have highlighted that frontline healthcare workers had lower secondary traumatization scores than non-frontline health workers and the general public in contrast to the findings of previous research on the SARS outbreak in the same area in Singapore (Chan and Huak, 2004). According to Barleycorn (2019) and Tan et al. (2020), these results may be due to the dedicated training and psychological support given to healthcare workers after the SARS outbreak and demonstrate the validity of policy strategies for prevention of stress in the psychological health field.

An analysis of 14 studies published from January to March 2020 aimed at investigating the stress experience of healthcare workers in facing COVID-19 shows that health workers experienced symptoms of depression and anxiety related to this stressful experience. Moreover, the severity of their symptoms was influenced by their age, gender, role, specialization, type of activity performed, and exposure to patients with COVID-19; however, prevention, resilience, and social support interventions mediated their response to stress (Bohlken et al., 2020). In a review of the literature, Spoorthy (2020) underlined that sociodemographic variables, such as age, gender, profession, and workplace, and psychological variables, such as poor social support and self-efficacy, affect the stress level experienced by health workers. In addition, COVID-19 emerged as an independent stress risk factor. Xiao et al. (2020) found that social support plays a role in reducing the anxiety levels in medical staff and increases their sense of self-efficacy.

According to Walton et al. (2020), the specific stressors that health workers face in the COVID-19 emergency are related to the organizational context. The challenges for medical staff include not only an increased workload but also a fear of infection, the need to work with new protocols that change frequently, and the use of PPE. In uncontrollable situations such as a pandemic, when specific action protocols are absent and limited resources are available, health workers must make individual decisions with a heavy burden of responsibility that may be contrary to their moral principles. For example, in the case of COVID-19, they may have to choose which patients to save because only a few places are available in intensive care. In this regard, Cai et al. (2020) showed that for a sample of 534 healthcare professionals who worked closely with COVID-19 patients in Hubei, the most stressful factors were the lack of protocols for the treatment of COVID-19, the scarcity of PPE, the exhausting work shifts, their concern about the risk of infection, and their exposure to the death and suffering of their patients. They also found that the support of superiors proved to be one of the most important motivational factors for medical staff, and the presence of clear guidelines and effective safety protocols were protective factors against the development of stress, in particular, for females. Further, Walton et al. (2020) identified the organizational stressors as the changes in work shifts, the prevalence of night shifts, an excessive workload, staff roles, autonomy, the lack of support from superiors, and the absence of adequate information and clear instructions. On the basis of these stressors, they estimated that 10% of the medical staff working on the front line of this pandemic are at risk of developing post-traumatic stress disorder (PTSD). In addition, limited resources, longer shifts, decreased hours of rest, and the occupational risks associated with COVID-19 exposure have increased the physical and mental fatigue, stress, anxiety, and burnout of these staff members (Sasangohar et al., 2020).

The loss of a social support network, which can be an important resilience factor, is another risk factor (Ozbay et al., 2007). In the COVID-19 emergency, healthcare and emergency workers have often experienced a separation from their affective links, either because of the restrictions on social contacts imposed by the lockdown or the fear of spreading the infection to their family members. To this must be added that although, at first, health workers received unanimous encouragement from the population, later, they also experienced demonstrations of stigma and isolation. Some studies have shown that being able to resort to their own social support network is a significant protective factor for health workers dealing with this emergency (Cai et al., 2020).

As Favretto (2005) stated, when individuals experience situations that go beyond their coping strategies, their vulnerability to, and risk of developing, psychopathological reactions increases. Studies conducted during previous epidemics, such as the SARS, MERS, and Ebola epidemics, converge in detecting how healthcare and emergency workers may experience extremely high levels of stress and even develop secondary traumatic stress or vicarious trauma. This trauma is defined as an experience of symptoms similar to those found in people with PTSD, such as in emergency nurses working with traumatized patients (Beck, 2011). Figley (1995) defined it as a form of stress that derives from the feelings of empathy experienced when helping traumatized people. The symptoms may include intrusive recurring thoughts, disturbed sleep, fatigue, physical symptoms, hyperarousal, increased stress response, anxiety, depression, and feeling emotional (Adriaenssens et al., 2012). Wolf et al. (2016) described how nurses may feel “overwhelmed,” and this condition becomes a source of moral distress that triggers feelings of powerlessness, guilt, fear, anger, and frustration.

The sense of frustration and impotence felt by nurses when they are unable to treat and save a patient has been highlighted as a risk factor for secondary traumatic stress in several studies (Missouridou, 2017). Avoidance and emotional numbing can become tools for self-protection from intrusive symptoms that exceed the personal tolerance level (Coetzee and Klopper, 2010; Mealer and Jones, 2013). Their frustration obviously intensifies on a patient’s death. The onset of PTSD in the health workers involved in treating MERS was also detected after the acute phase of the emergency was over, highlighting a risk not only in the immediate period but also in the medium-term period (Lee et al., 2018).

In reference to COVID-19, updated studies conducted on Chinese health workers have already highlighted the strong impact of the epidemic on the psychological health of doctors and nurses. Some studies have found that healthcare workers have high levels of anxiety, depression, insomnia, and distress (Lai et al., 2020; Li et al., 2020; Zhu et al., 2020). In particular, female professionals with more than 10 years of experience and previous psychiatric pathology present more risk factors of developing the symptoms of stress, anxiety, and depression (Lai et al., 2020; Zhu et al., 2020). Huang J. Z. et al. (2020) studied stress levels during the COVID-19 emergency in a sample of medical staff. They found that females showed higher levels of anxiety and PTSD than males did and that the levels were higher for nurses than for doctors. Moreover, Li et al. (2020) found that nurses had developed higher levels of vicarious trauma than those of the general population and that nurses who did not work closely with COVID-19 patients showed a more severe symptomatology, both physical and psychological, compared with their colleagues working on the frontline emergency services. In Italy, a study conducted on healthcare workers found that doctors and nurses developed high levels of stress and anxiety, greater than those developed by the general population, and that healthcare workers operating in the North, the area of Italy most affected by the virus, showed a more severe symptomatology (Simione and Gnagnarella, 2020). This study also confirmed that females tend to have a greater perception of the risk of infection, which increases their risk of developing the symptoms of anxiety and distress.

Because of their long, intense exposure to various stressors, it is important to note the nature of the coping strategies used by these healthcare and emergency workers in these situations and their effectiveness in terms of reducing and effectively coping with stress. Indeed, the effective management of stress levels in the acute/emergency phase could reduce the risk of developing long-term PTSD or other pathologies, such as anxiety and depression (Fullerton et al., 2004; Slottje et al., 2005; Argentero and Setti, 2011; Sakuma et al., 2015; Birinci and Erden, 2016; Li et al., 2017). Coping may be defined as a series of cognitive and behavioral efforts to manage specific internal or external issues that test or exceed individual resources (Lazarus and Folkman, 1984). A distinction can be made between problem-focused and emotion-focused coping strategies. The former is aimed at modifying and solving the stressful situation through active interventions. By contrast, emotion-focused coping is aimed at managing the emotions connected to the stressful event and regulating affective reactions, such as anxiety and the tension of response to stress, for example, by trying to avoid the threat (denial) or re-evaluating it (reappraisal).

The choice of coping strategies is influenced by the individual’s cognitive evaluation of the event, termed secondary evaluation, which involves estimating the resources available and the most effective strategies to deal with the situation (Lazarus and Folkman, 1984). A key element of this assessment is the extent to which the individual can maintain control over the outcome of the situation. The literature indicates that individuals apply dysfunctional coping when they face an uncontrollable event by responding primarily with a coping strategy focused on the problem, and conversely, when they face a controllable situation, they respond with coping strategies focused on emotions (Strentz and Auerbach, 1988; Vitaliano et al., 1990). A coping strategy may be defined as adaptive when the controllability of the stressful event corresponds with the choice of coping strategy: in this case, the subject will experience fewer symptoms related to stress (Park et al., 2001).

The strategies used to cope with trauma may differ among individuals, but they can also vary according to the profession and the features of the traumatic event (Nydegger et al., 2011). Individuals differ in their choice of coping strategies (Connor-Smith and Flachsbart, 2007), and factors related to the situation can also have a decisive influence on such choice (Brown et al., 2002). A few studies have considered the ways in which gender influences the perception of stress in emergency situations and the choice of coping strategy. These studies highlight that females tend to perceive events as more negative and uncontrollable and to resort more to coping strategies focused on emotions and avoidance, whereas males tend to resort more to applying problem-focused coping and to inhibiting emotions (Matud, 2004; Matud et al., 2015; Matud and Garcia, 2019).

The literature on the relationship between coping strategies and the stress levels of emergency workers has shown that the use of coping strategies focused on the problem usually tends to correlate with lower stress levels, both in healthcare workers (Watson et al., 2008; Howlett et al., 2015) and in other emergency workers, such as firefighters (Brown et al., 2002). However, a coping strategy frequently used by emergency workers is that of avoidance and minimization, and this strategy is associated with higher levels of stress (Brown et al., 2002; Chang et al., 2003; Kerai et al., 2017; Witt et al., 2018; Theleritis et al., 2020). Loo et al. (2016) found that in a group of emergency workers, avoidance as well as coping strategies focused on emotions were associated with the development of post-traumatic symptomatology. Rodríguez-Rey et al. (2019) revealed that among health workers working in a pediatric emergency department, approximately 30% of the variance in PTSD was explained by the frequent use of coping strategies focused on emotions and the infrequent use of those focused on the problem. In addition, Kucmin et al. (2018), who considered a sample of 440 paramedics, highlighted that the risk of developing PTSD symptoms was predicted by the use of coping strategies focused on emotions.

However, the literature does not offer unanimous results. Chamberlin and Green (2010) found that in a group of firefighters, all coping strategies actually correlated with high levels of stress: the authors explained this finding by suggesting that it is not the individual coping strategies that are maladaptive in themselves, but that greater effort is needed to adjust in stressful situations. By contrast, Young et al. (2014) indicated that firefighters use problem-focused coping strategies more often at the beginning of the operation and emotion-focused coping strategies more commonly in the phase of breakdown and fatigue. However, after the incident, they use both strategies (Young et al., 2014). A meta-analysis by Shin et al. (2014) highlighted that different coping strategies have different effects on work burnout: in particular, emotional stress and depersonalization are associated with the use of emotion-focused coping strategies, whereas professional ineffectiveness is associated with the use of problem-focused strategies.

Further, a few studies have investigated the coping strategies that emergency workers can use during health emergencies similar to COVID-19. Maunder et al. (2006) revealed that healthcare professionals who tended to apply dysfunctional coping strategies, based on avoidance, hostile comparison, or self-blame, tended to develop higher stress levels. Wong et al. (2005) highlighted that during the SARS epidemic, doctors and nurses tended to use different coping strategies. The doctors tended to turn more to action planning, but this strategy did not affect their stress level. Instead, their stress level was positively correlated with their use of coping strategies based on emotional outlets. By contrast, the nursing staff tended to resort more to behavioral disengagement and distraction strategies, which, however, correlated with higher levels of stress among them.

In this regard, during the MERS epidemic, hospital staff tended to adopt coping strategies related to the use of PPE and the adoption of all prevention measures, as well as social support, whereas the coping strategy that they adopted the least was that based on an emotional outlet (Khalid et al., 2016). A recent study on healthcare workers in Hubei, China, during the COVID-19 epidemic (Cai et al., 2020), yielded similar results: to reduce stress, the medical staff tended to rely on active coping strategies, such as using security protocols, practicing social isolation measures, and seeking support from family and friends, but they did not find it necessary to discuss their emotions with a professional. Huang L. et al. (2020) found that a sample of nurses working during the COVID-19 emergency presented greater emotional reactions and turned more to problem-focused coping compared with university nursing students. Emergency workers must have sufficient self-efficacy in terms of their coping skills to be able to manage and cope with stress levels. Self-efficacy in coping appears to be an effective protective factor in relation to stress levels and maladaptive responses (Chesney et al., 2006). Self-efficacy to cope with traumatic events has been effective in reducing the risk of developing PTSD (Bosmans et al., 2015).

## Materials and Methods

### Objectives

The main objective of this study is to identify the coping strategies activated by healthcare and emergency workers to deal with stress factors related to the COVID-19 emergency that may be associated with the risk of developing vicarious or secondary trauma. Few studies have considered both groups simultaneously when analyzing the strategies they have adopted to manage stress during the COVID-19 emergency. Hence, in this study, we are interested in detecting the similarities and differences in the approaches they adopted to manage their stress during the acute phase of the current pandemic According to Walton et al. (2020), the main acute stress reactions of emergency workers to emergency medical situations are emotional, cognitive, physical, and social reactions. Therefore, these factors were included in the questionnaire used in the present study. Moreover, reactions linked to stress factors for difficulties due to ineffective decision-making and dealing with stress were also considered (Chesney et al., 2006). In addition, fears regarding contracting the virus and infecting their own families because of COVID-19 were specifically considered (Du et al., 2020; Huang J. Z. et al., 2020; Ornell et al., 2020; Walton et al., 2020).

Based on results found in the literature, the specific objectives of this study are as follows:

(1) To examine the relationships between coping strategies, emergency stress, and secondary trauma in healthcare and emergency workers.
(2) To identify significant differences in stress factors, coping strategies, and secondary trauma between two groups—health workers and emergency workers.
(3) To analyze the predictive power of coping strategies on the various levels of stress.
(4) To analyze the predictive power of stress factors on the levels of arousal and intrusion of secondary trauma.
(5) To analyze the predictive power of coping strategies on the levels of arousal and intrusion of secondary trauma.

### Method

#### Participants

Participants were selected on a voluntary basis through a trasversal sampling in order to take a picture of the situation caused by the pandemic emergency. We used an internet platform to conduct the study and approached the participants using social media, dedicated mailing lists, and forums. Participants from all Italian regions completed the questionnaire online. The sample consists of 210 participants—90 males (42.9%) and 120 females (57.1%)—whose average age was 42.53 years (*SD* = 10.97; min 22 – max 67). Further, 52.9% of the sample were married, 10.6% were separated, and the remaining 36.5% were single. We selected various professional figures who had directly worked in various sectors during the COVID-19 emergency and who could be divided into two main groups. The first, the “Health Group,” consists of 121 participants (57.6%) who were healthcare workers: 57 doctors (50%), 47 nurses (37.3%), 9 psychologists (7.14%), and seven healthcare assistants (5.56%). Their average age was 42.13 years (*SD* = 11.35), and their average years of active professional service was 14.60 (*SD* = 11.56). The second, the “Emergency Group,” consists of 89 participants (42.4%): 48 emergency workers (53.9%), 21 firefighters (23.6%), and 20 Civil Protection staff (22.5%), whose average age was 45.43 years (*SD* = 10.19) and average years of service was 14.41 (*SD* = 11.89). There was an age difference between the two groups (*t* = −2.170; *p* <0.05), and the distribution of the gender variable differed between the two groups, with 41 males and 80 females in the Health Group and 49 males and 40 females in the Emergency Group (χ^2^ 9.38; *p* < 0.01). The study involved participants from the entire national territory, and their workplace could be divided as follows: 38, 36, and 26% were from North, Central, and South Italy, respectively. Further, 59% of the sample worked directly with COVID-19 patients and 24.8% worked in specific COVID-19 departments. Among the healthcare workers, 73% had worked in direct contact with COVID-19 patients, whereas among the emergency workers, only 33% had assisted these patients (χ^2^ 36.251; *p* < 0.01). In the present study, we included two variables, lack of necessary instructions and lack of PPE, in accordance with the findings in the literature on their impact on the stress reactions of healthcare and emergency workers during the COVID-19 pandemic. Among the participants, 62 and 45% of healthcare and emergency workers, respectively, did not have sufficient instructions to intervene (χ^2^ 2.441; *p* n.s.), and 57 and 52% of healthcare and emergency workers, respectively, lacked adequate PPE when working (χ^2^ 2.857; *p* n.s.).

#### Procedure

This study used an online questionnaire and was conducted during the lockdown period owing to the COVID-19 pandemic. The questionnaire had three parts: one each to collect online informed consent and baseline sociodemographic information, and one with an online series of questionnaires, as described in the next section. Participants’ anonymity was maintained in collecting the data. The institutional Ethics Committee approved all the procedures.

### Materials

We administered a series of questionnaires to evaluate the psychological stress and coping style of each participant. We included the following questionnaires.

#### Secondary Traumatic Stress Scale – Italian Version (STSS-I; Setti and Argentero, 2012)

This instrument’s 15 items enable verification of the presence of two symptoms of vicarious trauma, Intrusion and Arousal, and their relative frequency. The STSS was built on the basis of the conceptualization expressed in the DSM-5 (American Psychiatric Association, 2013) regarding the characteristic PTSD symptoms. In detail, the Arousal items describe situations characterized by anxiety, confusion, physical and psychological complaints, and agitation. Intrusion refers to the re-experiencing of the traumatic event—even if not directly suffered—through internal images and memories. Instructions for the STSS-I indicated that respondents should specify how frequently an item was true for them in the previous 4 weeks. The statements are evaluated on a 5-point scale (1 = never; 5 = very often) that provides scores for Intrusion (example items: “I thought about my work with victims when I didn’t intend to”; “Reminders of my work with clients upset me”) and Arousal (example items: “I had trouble concentrating”; “I was easily annoyed”; “I expected something bad to happen”; “I felt jumpy”). The reliability coefficients of the instrument are 0.87 and 0.81 for Arousal and Intrusion, respectively.

#### The Coping Self-Efficacy Scale – Short Form (CSES-SF; Chesney et al., 2006)

This is a 13-item measure of perceived self-efficacy for coping with challenges and threats. This measure focuses on the changes in individuals’ confidence in their ability to cope effectively, based on the self-efficacy theory (Bandura, 1997; Chesney et al., 2006). Participants were asked, “When things aren’t going well for you, or when you’re having problems, how confident or certain are you that you can do the following.” Then, they were asked to rate on an 11-point scale the extent to which they believed they could perform important behaviors for adaptive coping. The instrument yields three subscale scores: “problem-focused coping” (α = 0.91), “stop unpleasant emotions and thoughts” (α = 0.91), and “support” (α = 0.80). Anchor points on the scale are 0 (“cannot do at all”), 5 (“moderately certain can do”), and 10 (“certain can do”).

#### An Original Questionnaire on Stressful Factors

We constructed an ad hoc 7-item questionnaire that included Yes/No questions to detect stress factors identified by the literature, such as the availability of suitable equipment and the receipt of clear instructions during the COVID-19 coping experience. In this study, we present the results related to two of these items: “Instructions,” which refers to having received the necessary instructions to intervene, and “Equipment,” which refers to having PPE. Predictions of these factors have also been made in other studies (Du et al., 2020; Walton et al., 2020). In light of the relevance and specificity of the lack of clear information or instructions and adequate PPE in the management of COVID-19 in the Italian context, as well as the findings in other studies, we decided to focus attention on these two risk factors.

#### Emergency Stress Questionnaire (ESQ; Vagni et al., 2020)

Our analysis of the literature revealed that in situations in which they have to cope with a pandemic, several factors may affect the stress of medical staff and emergency healthcare workers and that COVID-19 represents an independent specific stressor (Spoorthy, 2020). These stress factors have been identified as frequently affecting healthcare and emergency workers in emergency situations and leading to physical, emotional, cognitive, decision-making, relational, and organizational stress (Del Missier et al., 2008; Sbattella, 2009; Argentero and Setti, 2011; Fraccaroli and Balducci, 2011; Bellelli and Di Schiena, 2012; Du et al., 2020; Walton et al., 2020). Focusing on the specificity of the COVID-19 epidemic, items have been constructed regarding the fears of contracting the infection and of infecting colleagues or family members (Walton et al., 2020), since COVID-19 represents a factor of independent stress (Spoorthy, 2020) that has great impact (Huang J. Z. et al., 2020). Consequently, we constructed the ESQ consisting of 33 items assessed on a 5-point Likert scale, with scores ranging from 0 (not at all) to 4 (very much), grouped into six scales. The participants were asked to indicate how often they experienced certain emotions and thoughts while performing intervention and emergency activities during the COVID-19 pandemic.

The scales correspond to the factors identified and confirmed by factorial analysis through an analysis of the main components with orthogonal rotation of factors (varimax). The number of factors to be extracted was initially verified through the unit’s largest eigenvalue criterion and, subsequently, by the scree test. The ESQ is based on six scales:

(1) Organizational–Relational Stress: measures the stress levels related to the organizational context, relationships with colleagues, and social support (consisting of eight items: 7, 10, 13, 14, 15, 16, 19, and 23);
(2) Physical Stress: composed of five items describing symptoms of physical fatigue (11, 12, 18, 20, and 32);
(3) Inefficacy Decisional Stress: consists of five items that analyze decision-making aspects and the possibility to act, which are related to the level of self-efficacy (22, 25, 27, 28, and 29);
(4) Emotional Stress: comprises six items that indicate the participant’s emotional reactions (1, 2, 3, 4, 6, and 26);
(5) Cognitive Stress: consists of four items on the cognitive aspects of stress (5, 17, 21, and 24);
(6) COVID-19 Stress: comprises five items regarding worries related to the COVID-19 emergency (8, 9, 30, 31, and 33).

The ESQ demonstrated good internal consistency (α = 0.93) overall and for each individual scale: Organizational–Relational Stress (α = 0.71), Physical Stress (α = 0.82), Inefficacy Decisional Stress (α = 0.80), Emotional Stress (α = 0.86), Cognitive stress (α = 0.72), and COVID-19 Stress (α = 0.80).

### Statistical Strategy Explanation

First, we performed Pearson’s correlation analyses to identify the associations between the variables for the two groups that we considered in this study. Subsequently, we checked for significant differences between the two groups as their stress levels, coping strategies, and secondary trauma. We used hierarchical linear regression models to verify the predictive effect of the risk factors (lack of adequate information and PPE) on the different stress levels (in step 1). Then, we verified the protective effect of the coping strategies (in step 2). The models were controlled for age, gender, and group. Lastly, we used hierarchical regression models to verify the predictive effect of stress factors on the components of secondary trauma. The models were controlled for age, gender, and group.

## Results

First, we conducted correlational analyses and comparisons of averages on the reference sample. Table 1 shows the correlations between the scales of the ESQ and the other instruments.

**TABLE 1:** Intercorrelations of STSS-I, ESQ, and CSES-SF for Health (above diagonal), and Emergency (below diagonal) Groups (*n* = 210).

	**STSS-I**		**ESQ**						**CSES-SF**		
	**Arousal**	**Intrusion**	**Organizational _relational stress**	**Physical stress**	**Inefficacy decisional stress**	**Emotional stress**	**Cognitive stress**	**COVID-19 stress**	**Focused problem**	**Stop emotion_ thought**	**Support**
**STSS-I**											
Arousal		0.491***	0.196*	−0.176*	0.119	–0.022	0.259**	0.179*	0.127	0.044	–0.136
Intrusion	0.463***		0.136	–0.065	0.240*	–0.040	0.190*	0.197*	–0.017	–0.064	–0.140
**ESQ**											
Organizational_ relational stress	0.264**	0.066		0.299**	0.253**	0.315**	0.346***	0.569***	−0.258**	−0.227*	−0.192*
Physical stress	0.013	−0.160*	430***		0.183*	0.476***	0.406***	0.328**	−0.448***	−0.324**	−0.399***
Inefficacy decisional stress	0.170*	0.098	495***	0.251**		0.246*	0.322**	0.391***	–0.003	–0.110	0.036
Emotional stress	0.221*	0.021	483***	0.405***	0.365***		0.481***	0.398***	−0.384***	−0.398***	–0.158
Cognitive stress	0.366***	0.205*	513***	267**	0.391***	0.386***		0.418***	−0.279**	−0.292**	–0.166
COVID-19 stress	0.218**	–0.051	277**	452***	0.303***	0.464***	0.277**		−0.231*	−0.278**	−0.219*
**CSES-SF**											
Focused problem	–0.037	−0.157*	–0.122	−0.183*	–0.006	–0.139	–0.127	–0.016		0.487***	0.364***
Stop emotion_ thought	−0.325***	−0.292**	−0.346***	−0.194*	–0.120	−0.256**	−0.334***	–0.095	0.451***		0.419***
Support	−0.176*	−0.159*	–0.145	–0.145	0.096	–0.108	–0.084	0.005	0.270**	0.435***	

*p < 0.05, **p < 0.01, ***p < 0.001; STSS-I, Secondary Traumatic Stress Scale - Italian Version; ESQ, Emergency Stress Questionnaire; CSE-SDF, The Coping Self-Efficacy Scale – Short Form.

Preliminary comparisons were made through the Student’s *t*-test between the Health Group and the Emergency Group in relation to the ESQ, CSES-SF, and STSS-I scores. Table 2 shows the comparison between the two groups.

**TABLE 2:** Differences in STSS-I, ESQ, and CSES-SF between Health and Emergency Groups (*n* = 210).

	**Health group**	**Emergency group**		
	**Mean (*SD*)**	**Mean (*SD*)**	***t*-value**	**Cohen’s *d***
**ESQ**				
Organizational_ relational stress	22.69 (4.43)	19.43 (3.62)	5.69***	0.81
Physical stress	10.29 (3.13)	8.09 (4.60)	3.19**	0.45
Inefficacy decision	14.45 (3.13)	12.79 (3.05)	3.84***	0.54
Emotional stress	14.17 (3.48)	10.45 (3.16)	7.95***	1.12
Cognitive stress	8.88 (2.89)	6.08 (2.53)	7.30***	1.03
COVID-19 stress	15.54 (3.67)	12.74 (4.17)	5.37***	0.71
**CSES-SF**				
Focused problem	36.69 (6.76)	37.65 (6.57)	–1.04	0.14
Stop emotion_ thought	32.50 (10.59)	36.40 (9.00)	−2.81**	0.40
Support	21.25 (5.88)	21.09 (6.54)	0.183	0.03
**STSS-I**				
Arousal	26.33 (4.97)	23.30 (5.51)	4.15***	0.58
Intrusion	15.38 (5.22)	14.55 (5.32)	1.23	0.16

**p < 0.01, ***p < 0.001; STSS-I, Secondary Traumatic Stress Scale - Italian Version; ESQ, Emergency Stress Questionnaire; CSE-SF, The Coping Self-Efficacy Scale – Short Form.

As shown in Table 2, significant differences emerged between the two groups in relation to their Stress and Arousal levels. The results indicate higher levels of both for the Health Group, and that emergency workers turn more to the Stop Unpleasant Emotions and Thoughts strategy. Further, we performed comparisons with reference to the gender variable to detect differences in the levels of stress factors, coping strategies, and secondary trauma. Females reported significantly higher Physical Stress than males did (Females: *M* = 10.90; *SD* = 4.83; Males: *M* = 7.30; *SD* = 4.57; *t* = 5.47; *p* < 0.001), as well as Emotional Stress (Females: *M* = 13.30; *SD* = 3.68; Males: *M* = 11.64; *SD* = 3.80; *t* = 3.18; *p* < 0.01) and COVID-19 Stress (Females: *M* = 14.93; *SD* = 3.68; Males: *M* = 13.58; *SD* = 4.22; *t* = 2.48; *p* < 0.05). No gender difference emerged in coping strategies and secondary trauma. Within the Health Group, there were significant differences regarding Inefficacy Decisional Stress (*F* = 3.68; *p* < 0.05; Doctor *M* = 14.51; *SD* = 2.89; Psychologist *M* = 11.11; *SD* = 2.15; average difference = 3.40; *p* < 0.05); and COVID-19 Stress (*F* = 3.57, *p* < 0.05; Nurse *M* = 16.19; *SD* = 3.47; Doctor *M* = 14.30; *SD* = 3.61; difference = 1.89, *p* < 0.05). Within the Emergency Group, there were no differences in levels of stress and secondary trauma or coping strategies. Moreover, we found similar correlations between the two groups for the Stop Unpleasant Emotions and Thoughts strategy and the stress factors, whereas for the other two coping strategies, we found a different association, particularly for the Emergency Group. However, the *t*-test comparisons highlight differences only at the level of the Stop Unpleasant Emotions and Thoughts strategy. Given the findings of the preliminary analyses, we considered it necessary to include the age, gender, and group variables to test the predictiveness of the coping strategies on the participants’ stress levels.

To test the predictive effect of the coping strategies on various levels of stress, hierarchical regression was conducted. Considering the Age and Gender differences within the groups, we included these variables in all models together with the Group variable (Health vs. Emergency) and the “Instructions” and “Equipment” variables. The models generated by assuming the ESQ scales as dependent variables are shown in Table 3. Regarding the coping strategies, we observed an important effect of the Stop Unpleasant Emotions and Thoughts Coping strategy on all the stress scales, except for Physical Stress where the effect of the Focused Problem Coping strategy is recorded.

**TABLE 3:** Hierarchical regressions on ESQ scales (*n* = 210).

	**Organizational_ relational stress**		**Physical stress**		**Inefficacy decision stress**		**Emotional stress**		**Cognitive stress**		**COVID-19 stress**	
	***B***	**Exp (*B*)**	***B***	**Exp (*B*)**	***B***	**Exp (*B*)**	***B***	**Exp (*B*)**	***B***	**Exp (*B*)**	***B***	**Exp (*B*)**
**Model 1**												
Age	–0.042	–0.104	–0.072	−0.157*	–0.023	–0.081	–0.062	−0.177**	–0.027	–0.096	0.001	0.002
Gender^1^	0.407	0.046	3.521	0.347***	–0.892	−0.138*	1.105	0.144*	0.272	0.044	0.871	0.109
Health/emergency group	–2.001	−0.225***	–0.672	–0.066	–1.389	−0.215**	–2.884	−0.375***	–2.164	−0.349***	–2.399	−0.300***
Instructions^2^	3.382	0.375***	1.623	0.158*	1.092	0.167*	1.150	0.147*	1.563	0.249**	0.464	0.057
Equipment^3^	0.756	0.086	1.283	0.127	1.200	0.188*	1.094	0.143*	0.454	0.074	1.587	0.200**
	*R*^2^ = 0.313		*R*^2^ 0.225		*R*^2^ 0.185		*R*^2^ 0.333		*R*^2^ 0.293		*R*^2^ 0.186	
	*F* = 18.560***		*F* = 11.855***		*F* = 9.258***		*F* = 20.352		*F* = 16.892***		*F* = 9.330***	
**Model 2**												
Age	–0.020	–0.051	–0.047	–0.102	–0.010	–0.046	–0.041	−0.117*	–0.009	–0.032	0.015	0.042
Gender^1^	0.260	0.029	3.254	0.320***	–0.804	–0.125	0.940	0.122*	0.158	0.026	0.756	0.095
Health/emergency group	–1.750	−0.197**	–0.586	–0.058	–1.127	−0.175*	–2.705	−0.351***	–1.960	−0.316***	–2.268	−0.283***
Instructions^2^	3.133	0.348***	1.244	0.121	1.241	0.190*	0.898	0.115	1.379	0.220**	0.279	0.034
Equipment^3^	0.834	0.095	1.316	0.131	1.340	0.209**	1.157	0.152*	0.527	0.086	1.628	0.205**
Focused problem	0.040	0.008	–0.125	−0.166*	0.028	0.059	–0.040	–0.070	–0.009	–0.020	–0.017	–0.028
Stop emotion_ thought	–0.108	0.055	0.054	–0.109	–0.061	−0.192*	–0.080	−0.211**	–0.083	−0.274***	–0.061	−0.154*
Support	–0.108	0.055	0.032	0.039	0.105	0.202**	0.040	0.064	0.044	0.088	0.022	0.034
	*R*^2^ 0.359		*R*^2^ 0.270		*R*^2^ 0.226		*R*^2^ 0.379		*R*^2^ 0.352		*R*^2^ 0.208	
	*F* = 14.041***		*F* = 9.273***		*F* = 7.352***		*F* = 15.346***		*F* = 13.634***		*F* = 6.596***	

*p < 0.05, **p < 0.01, ***p < 0. 001; ^1^Gender (1 = male; 2 = female); ^2^Instructions (1 = yes; 2 = no); ^3^Equipment (1 = yes; 2 = no); CSES-SF Scales, Focused Problem; Stop Emotion_Thought; Support.

As shown in Table 1, significant negative associations between stressors and secondary trauma were found for both groups. The hierarchical regression models of stress scales were analyzed for the Arousal and Intrusion levels of secondary trauma. The models included the Age, Gender, Health/Emergency Group variables, and the ESQ scales. The results are shown in Table 4.

**TABLE 4:** Hierarchical regressions on Arousal and Intrusion (*n* = 210).

	**Arousal**		**Intrusion**	
	**Exp(*B*)**	***B***	**Exp(*B*)**	***B***
**Model 1**				
Age	0.034	0.070	0.004	0.008
Gender^1^	0.026	0.002	–1.193	–0.113
Health/emergency group	–3.126	−0.287***	–1.096	–0.103
	*R*^2^ 0.082		*R*^2^ 0.018	
	*F* = 6.062**		*F* = 1.270 n.s.	
**Model 2**				
Age	0.034	0.069	–0.002	–0.004
Gender^1^	0.736	0.067	–0.264	–0.025
Health/emergency group	–0.911	–0.084	0.044	0.004
Organizational_ relational stress	0.205	−0.165*	0.052	0.043
Physical stress	–0.303	−0.283***	–0.225	−0.216*
Inefficacy decisional stress	0.020	0.012	0.189	0.115
Emotional stress	–0.018	–0.012	–0.120	–0.087
Cognitive stress	0.564	0.316***	0.067	0.050
COVID-19 stress	0.196	0.144	0.427	0.249***
	*R*^2^ 0.247		*R*^2^ 0.102	
	*F* = 7.216***		*F* = 2.534**	

*p < 0.05, **p < 0.01, ***p < 0.001; ^1^Gender (1 = male; 2 = female).

The same regression models were generated by including coping strategies as predictors and were analyzed by Age, Gender, and Health/Emergency Group. Compared with Arousal, the Health/Emergency Group and Stop Unpleasant Emotions and Thoughts are predictive (*R*^2^ 0.138; *F* = 5.343; *p* < 0.001; Beta −0.264^∗∗∗^; Beta −0.207^∗^, respectively). Compared with Intrusion, only the Stop Unpleasant Emotions and Thoughts variable (*R*^2^ 0.065; *F* = 2.347; *p* < 0.05; Beta −0.182^∗^) assumes significance.

## Discussion

The results of this study show that healthcare and emergency workers both experienced high stressors during the COVID-19 epidemic, exposing them to the risk of developing secondary trauma (Dominguez-Gomez and Rutledge, 2009; Argentero and Setti, 2011; Adriaenssens et al., 2012; Duffy et al., 2015; Aisling et al., 2016; Morrison and Joy, 2016; Wolf et al., 2016; Roden-Foreman et al., 2017; Lai et al., 2020; Li et al., 2020; Zhu et al., 2020). We found significant differences between the two groups regarding their reactions and their levels of organizational, physical, and relational stress, their sense of decision-making, and their emotional and cognitive ineffectiveness. Compared with emergency workers, healthcare workers had higher stress levels, leading them to perceive more serious tensions and difficulties in teamwork, physical fatigue, somatic illnesses, irritability, and difficulty in maintaining control over the situation, in taking decisions, and in predicting the consequences of their actions. Higher levels of stress have been reported related to the fears of contracting COVID-19 and of infecting family members. In line with other studies, we found that the COVID-19 emergency led health workers, in particular, to perceive specific stress factors that affected the organizational area, with consequences in terms of tension in teamwork and a sense of ineffectiveness since they had to intervene without sufficient tools and resources. They also experienced deep emotional reactions of anger, powerlessness, and frustration with inevitable cognitive stress, in terms of increased arousal levels. Many of the healthcare workers also developed physical stress, due not only to the lack of sleep but also to the possible forms of somatization of the psycho-emotional tension they perceived (Sasangohar et al., 2020; Walton et al., 2020).

The differences recorded between the two groups in stress levels may be explained by taking into account, for example, the fact that the Emergency Group perceived their intervention with a greater sense of continuity in their usual procedures compared with the Health Group. The former performed their usual activities on the organizational, cognitive, and procedural levels, although with greater levels of safety and self-protection and a greater frequency of interventions. Conversely, the Health Group had to reorganize aspects such as departments, teams, and shifts to cope with the emergency, which thus involved making radical changes. In addition, the Health Group helplessly witnessed a large number of deaths of their patients and had to make decisions in conflict with their moral sense and in situations of insecurity and unpredictability regarding the consequences of their actions (Cai et al., 2020; Walton et al., 2020). However, in terms of physical stress, there was no predictive effect of the group, which indicates that the Health and Emergency Groups were both exposed to very similar physical stressors.

It is important to consider the significant impact of the gender variable. According to other studies, females developed a greater reaction of physical and emotional stress and the sense of decision-making ineffectiveness than did males (Lai et al., 2020; Zhu et al., 2020). In fact, females apparently tend to perceive events as more negative and uncontrollable, and thus suffer higher levels of stress. Further, females tend to resort to coping strategies focused on emotions, which tend to be less effective in emergency situations (Matud, 2004; Matud et al., 2015; Matud and Garcia, 2019). However, in the present study, these gender differences did not have an impact in terms of psychopathological or specific maladaptive consequences, and coping strategies. In fact, females and males perceived a similar sense of efficacy/ineffectiveness in dealing with stressful situations and had similar scores on the secondary trauma scale. The results shown in Table 3 also indicate that predictive impact is also assumed by the lack of adequate instructions and knowledge about the emergency and the lack of necessary PPE. In particular, for the Health Group, the lack of necessary instructions on how to conduct quick interventions affected almost all stressors, leading to tensions or conflicts within the team, difficulty in making decisions, irritability, anger, and frustration.

Above all, the lack of PPE affected the sense of making the right decisions, the emotional sphere and, most importantly, the fear of contracting the virus or of transmitting it to their families. These results converge with those of other studies that have highlighted that the lack of adequate and specific information and of equipment for healthcare staff in dealing with COVID-19 affected their self-efficacy and the factors protecting them from stress, thus increasing their fear of contracting an infectious disease and causing them greater emotional, decisional, and physical stress. Conversely, the professionals who were provided with the necessary knowledge and equipment were more resilient during the emergency response (Du et al., 2020; Huang J. Z. et al., 2020; Ornell et al., 2020; Walton et al., 2020). The lack of specific equipment and instruments in emergency situations along with the risk of infection increases the feeling of poor control, leading to cognitive and emotional stress and a sense of ineffectiveness (Placentino and Scarcella, 2001; Walton et al., 2020). Higher levels of stress were found in the Health Group than in the Emergency Group because of the absence of PPE, the risk of infection from the virus, and the lack of necessary instructions or prompt information (Cai et al., 2020). The incidence of these variables is contained and limited by the use of coping strategies.

The coping strategy that assumes a predictive effect, reducing stress levels, is to block those negative or unpleasant emotions and thoughts associated with the risk of developing secondary trauma. In fact, the use of the Stop Unpleasant Emotions and Thoughts strategy reduces the Arousal and Intrusion levels of the secondary trauma. The effectiveness of this strategy in reducing the Arousal levels appeared to be greater in the Health Group. As Fraccaroli and Balducci (2011) suggested, in situations of high emergency stress, healthcare workers and emergency workers may have a deficit in the cognitive process of emotions, thus failing to identify their emotional reactions, which tends to be associated with maladaptive behaviors. The lack of a complete recognition of one’s unpleasant emotions, which tends to be denied and dismissed as a coping strategy, would explain the greater predictive impact of cognitive stress and physical stress on post-traumatic arousal compared with emotional stress.

Further, the results of this study highlight that the Stop Unpleasant Emotions and Thoughts strategy has an inhibitory and therefore effective and highly significant impact on the stress levels and the components of secondary trauma, unlike the problem-focused and social support strategies. The literature points out that the avoidant matrix coping strategies tend to present themselves when healthcare and emergency workers experience a condition of fatigue and exhaustion, and this would explain the presence of the greater acute stress responses in healthcare workers (Maunder et al., 2006; Young et al., 2014).

The results of this study show that the problem-focused coping strategy (the strategy most frequently used in the Health Group in line with the finding of Huang L. et al., 2020) in this emergency situation did not appear to demonstrate protective efficacy. This is likely to be because the workers were dealing with an emergency that was not yet fully understood and the therapeutic and treatment procedures were not fully known. Moreover, the supply of PPE was scarce, especially in the first few weeks of the COVID-19 emergency in Italy, in all hospitals (e.g., a lack of respirators and insufficient number of resuscitation beds), which meant that the level of protective efficacy of this strategy may have been lower than the stress levels.

In other words, emergency workers, although task-oriented, were faced with a problem that was not fully understood, and in the absence of PPE, perceived poor self-efficacy in terms of trust and belief in their ability to organize and make effective decisions. The strategy that ensured optimal levels of self-efficacy was the one that allowed negative thoughts and emotions associated with the epidemic to be removed from consciousness, which was also found to have a protective function against the risk of developing traumatic symptoms.

The government lockdown and the consequent restriction of visits outside the working environment limited the use of coping strategies involving social support, family, and friends, implying a greater use of emotional and cognitive avoidance methods to deal with anguished thoughts, intrusive memories, and the constant vision of corpses or the seriously ill. In this regard, the Health Group appears to have developed a greater secondary trauma arousal than the Emergency Group. By contrast, the latter appears to have developed more aspects of intrusiveness related to secondary or vicarious trauma than the Health Group (see Table 2).

Since they were interviewed during the COVID-19 emergency, the healthcare and emergency workers who participated in the present study do not appear to have developed a complete secondary trauma. This may explain the prediction of the stress factors on arousal and not on intrusion. In other words, these individuals were interviewed while the emergency was still in the acute phase and before a structuring of answers in a psychopathological sense could be performed. Therefore, performing a follow-up study would be interesting. PTSD can take several months to fully emerge, and its stabilization can depend on the individual’s internal as well as external factors.

Because they blocked negative emotions and unpleasant memories, the healthcare and emergency workers’ arousal appears to be mainly due to, at least in the full phase during the epidemiological emergency, the factors of a cognitive matrix, linked with the difficulty of focusing on and identifying the most appropriate intervention strategies, leading them to experience regret, disappointment, and both physical and relational tension. The health workers apparently blocked the emotional aspects related to pain, impotence, and guilt, which allowed them to continue their work. In an emergency phase that is still active, and a few weeks after the start of the pandemic, it is possible to detect high arousal and a lower level of intrusiveness of stressful or traumatic events. This condition may be more likely if the blocking of negative emotions and intrusive thoughts linked to one’s personal experience intervenes as a coping strategy. Low perceptions of self-efficacy regarding coping has been found to be a predictor of PTSD in other studies (Benight and Harper, 2002; Bosmans et al., 2015).

In emergency situations, high stress can cause emergency workers to experience impotence, breathlessness, cognitive difficulties, and difficulties in decision-making and managing emotional reactions along with a prevalence of feelings of anger, as recorded in this study. If the lack of adequate knowledge and of PPE are added to these factors, even professional experts may perceive a loss of self-efficacy in coping and, simultaneously, experience an inability to orient their skills more effectively, thus developing maladaptive responses.

### Limitations

This study has several limitations. The first is the limited sample size. The second is that our study involved participants in the very midst of the COVID-19 emergency, which means that the level of stress in healthcare workers may have been more severe and acute. Moreover, the long-term psychological implications for the healthcare and emergency population should be investigated for the presence of a full secondary trauma. Therefore, a large-sized longitudinal study is called for to further explore the pathogenesis of vicarious traumatization. The third is that participants were not selected based on whether they had existing psychological problems. In proposing the hypothesis of this study, we anticipated that we would be able to discover the relationships between coping strategies, emergency-related stress, and secondary trauma in healthcare and emergency workers and commenced our investigation by assuming that the impact of stress can provoke psychological consequences in emergency situations. In future work, this assumption could be tested to verify whether an emergency situation has a different impact on workers who have already experienced psychological problems.

## Data Availability Statement

The raw data supporting the conclusions of this article will be made available by the authors, without undue reservation, to any qualified researcher.

## Ethics Statement

The studies involving human participants were reviewed and approved by Comitato Etico per la Sperimentazione Umana – CESU of the University of Urbino. The patients/participants provided their written informed consent to participate in this study.

## Author Contributions

MV, TM, VG, and DP: conceptualization, writing – original draft preparation, and writing – review and editing. MV, TM, and VG: methodology and investigation. MV and TM: formal analysis and data curation. TM and VG: visualization. MV, TM, and DP: project administration. All authors contributed to the article and approved the submitted version.

## Conflict of Interest

The authors declare that the research was conducted in the absence of any commercial or financial relationships that could be construed as a potential conflict of interest.
